# Plasma Homocysteine, Folic Acid and Vitamin B12 in Abruptio Placentae: A Cross-Sectional Study of Their Role and Feto-Maternal Outcome

**DOI:** 10.7759/cureus.35664

**Published:** 2023-03-01

**Authors:** Seema Meena, Harsha S Gaikwad, Banashree Nath

**Affiliations:** 1 Obstetrics and Gynaecology, Vardhman Mahavir Medical College and Safdarjung Hospital, New Delhi, IND; 2 Obstetrics and Gynaecology, All India Institute of Medical Sciences, Rae Bareli, IND

**Keywords:** pregnancy outcome, abruptio placentae, vitamin b12, folic acid, homocysteine

## Abstract

Background

Out of the many causes of abruptio placentae, the micronutrient association with its occurrence and severity has not been researched extensively till now. We aim to measure the serum levels of homocysteine, folic acid and vitamin B12 in patients with abruptio placentae in the third trimester of pregnancy and compare the levels with those without the complication. We also propose to compare the feto-maternal outcome between the groups.

Methods

The cross-sectional study was undertaken in 50 pregnant women with abruption before or during delivery and 50 controls with uncomplicated pregnancy over 28 weeks of gestation. Serum levels of homocysteine, folic acid, and vitamin B12 were determined and feto-maternal outcome was compared between the groups

Results

Mean age of the cases and controls are 26.82 ± 5.5 and 28.82 ± 4.88 years respectively. Obstetric characteristics have significant difference between the groups in terms of gravidity, mode of delivery, timing of delivery, proportion of stillbirths and blood transfusion. The mean concentration of homocysteine and vitamin B12 between the groups also have a significant difference . The serum level of homocysteine is significantly correlated with serum vitamin B12 level (Pearson correlation= -0.601, P=0.000). However, folic acid concentration between the groups remains comparable.

Conclusion

Hence we conclude that vitamin B12 and homocysteine are significant determinants of abruptio placentae in pregnant women. Supplementation with the vitamin in the high-risk Indian population can avert a number of obstetric complications occurring due to raised homocysteine.

## Introduction

Placental abruption is defined as premature separation of the placenta from the decidua before the end of second stage of labour. It can be either revealed with bleeding insinuating between membranes and uterus ultimately escaping through the cervix to cause external haemorrhage or it can be concealed with blood [1]. It is a clinical diagnosis, typically characterized by vaginal bleeding, abdominal pain, uterine contractions and/or tenderness, and sometimes non- reassuring foetal monitoring associated with an increase in the risk of stillbirth, preterm delivery, haemorrhage, need for hysterectomy, DIC and death [2]. Among the several factors playing a role in its causation, raised homocysteine levels have been associated with this obstetric complication.

Homocysteine is a naturally occurring amino acid, derived from demethylation of methionine requiring folate, vitamin B12, B6 as a co-enzyme. It is metabolized in the body to either cysteine using pyridoxine (vitamin B6) or it can be recycled to methionine using folic acid and methyl cobalamin (vitamin B12) as co-factors [3]. The increase in plasma total homocysteine can occur either from deficient folate and vitamin B12 status or due to causes related to nutritional, genetic, physiological and pathological factors. MTHFR gene or cystathionine-beta synthase mutation can cause an increase in serum homocysteine levels which defines the close association between folic acid, vitamin B12 and homocysteine [4]. MTHFR gene encodes the methylene tetrahydrofolate reductase (MTHFR), a rate-limiting enzyme that catalyses the conversion of 5,10-methylenetetrahydrofolate to 5-methylytetrahydrofolate. The product is a co-substrate for remethylation of homocysteine to methionine [5]. The raised homocysteine can eventually increase the risk of vascular abnormalities through oxidative stress leading to endothelial cell dysfunction. Placental function in pregnancy is also affected causing diverse adverse outcomes linked to placental insufficiency, which includes preeclampsia, spontaneous abortion, abruptio placentae, intrauterine growth restriction, recurrent pregnancy loss and preterm birth. Folic acid deficiency too has been implicated as a risk factor for abruptio placentae. The increased risk of abruptio placentae has been linked to polymorphisms in folate-related genes, suggesting that even a mild dysfunction in folate metabolism might predispose to adverse pregnancy events [6].

There are few studies in India that explore the effects of homocysteine, folic acid and vitamin B12 on the occurrence of abruption. Hence, the present study was designed to estimate the serum levels of homocysteine, folic acid and vitamin B12 in patients with abruptio placentae in the third trimester of pregnancy and compare the levels with those without the complication. We also propose to compare the feto-maternal outcome between the groups.

## Materials and methods

This was a single-centric hospital-based cross-sectional study conducted in the department of Obstetrics and Gynaecology at Vardhman Mahavir Medical College and Safdarjung Hospital for a period of 20 months. Study population comprised pregnant women with abruption attending ward and labour room of the department of Obstetrics and Gynaecology, satisfying inclusion criteria, during a period of 20 months from August 2018 to March 2020. A written and informed consent was taken from all the enrolled women in a language well understood by them. Institutional Ethics Committee (IEC) approval (approval IEC/VMMC/SJH/Thesis/October/2018-203) was obtained before starting the study. All the information procured from the study was kept confidential and used for academic purposes only.

Study population

The study population included all women diagnosed with abruption placentae either clinically or through imaging by ultrasound at gestational age of more than 28 weeks, before or during delivery. All delivered women whose placenta showed retroplacental haematoma/clots were also included in the study. Controls consisted of women with uncomplicated pregnancy delivering either spontaneously or after induction of labour. Women diagnosed with placenta previa, premature rupture of membranes, multiple pregnancy, uterine leiomyomas with a diameter >5cm, genital tract malignancy, polyhydramnios, medical co-morbid condition like anaemia, diabetes mellitus, hypertension, repeated miscarriage, preterm labour, smoking and those taking vitamin B12, folic acid, antifolate drugs or steroids were excluded from the study. Foetal growth and well-being was monitored by NST, BPP and Doppler velocimetry. Steroid for foetal lung maturation was given in women with pregnancy less than 34 weeks of gestation. Plan for timing and mode of delivery was done according to institutional protocol. Labour was monitored with partograph. Any complication during intrapartum and postpartum period was recorded. Status of the baby after birth including Apgar score at one and five minutes, birth weight and livebirth/stillbirth were recorded. Blood transfusion in both cases and controls was recorded.

Study procedure

Under aseptic precautions, 5ml blood was collected from antecubital vein of the subjects after six to eight hours of fasting, and samples were refrigerated until tested. In women who were diagnosed with abruption during delivery, blood was collected within 24hrs of delivery with maintenance of the fasting status of patient. Samples were centrifuged to separate cells and plasma. Following investigations were performed in all patients complete blood count, KFT, LFT, coagulation profile, urine albumin, serum TSH, serum levels of homocysteine, folic acid, vitamin B12 using commercially available kits.

Homocysteine, folic acid and vitamin B12 estimation

Homocysteine, folic acid and vitamin B12 estimation was done by enzymatic assay using ADVIA Centaur VB12, FOL and HCY assay kit (Tarrytown, NY, USA). The kits use a double-antibody sandwich enzyme-linked immunosorbent one-step process (ELISA) to assay the level of homocysteine, folic acid and vitamin B12. The kits have enzyme wells which are pre coated with antibody to the respective micronutrient to be measured to which standard test sample and horseradish peroxidase (HRP) labelled micronutrient antibodies are added followed by incubation for 60 minutes at 37 degree Celsius. Then the plate was washed five times and chromogen A and B solution was added followed by incubation for 15 minutes at 37 degree Celsius. On adding chromogen solution A and B, the colour of the liquid changes into blue and the reaction with the acid causes the colour to become yellow. The depth of the colour and the concentration of the micronutrient are positively correlated.

Normal range of serum homocysteine is 3.2-21.4 micromole/L. Normal range of serum folic acid is 1.4- 20.7 ng/ml. Normal range of serum vitamin B12 is 99-526 pg/ml.

Sample size

Mujawar et al. [7] found mean plasma homocysteine level of 16.4 ± 3.26 micromole/L in 50 patients with pre-ecclampsia. Taking this value as reference at 5% level of significance and 80% power of study with equal proportion of cases and controls, the minimum required number of cases and controls to detect a difference of 5 micromole/L between them is 34 patients. To reduce the margin of error, number of cases and controls was taken to be 50.

Statistical analysis

Categorical variables were presented in number and percentage (%) and continuous variables were presented as mean ± SD and median. Normality of data was be tested by Kolmogorov-Smirnov test. If the normality is rejected then non parametric tests were used. Quantitative variables were compared using Unpaired t-test/Mann-Whitney test (when the data sets were not normally distributed) between the groups. Qualitative variables were compared using Chi-Square test/Fisher’s exact test. The data was entered in Excel spreadsheet (Microsoft, Redmond, WA, USA) and analysis was done using Statistical Package for Social Sciences (SPSS) version 21.0 (IBM Corp., Armonk, NY, USA).

## Results

The study was undertaken in 50 pregnant women with abruption and 50 controls with uncomplicated pregnancy. The demographic and obstetric characteristics of both groups of women are presented in Table 1. Mean age of the cases and controls are 26.82 ± 5.5 and 28.82 ± 4.88 years respectively. Demographic characteristics of the groups showed no significant difference between the groups. Obstetric characteristics however have significant differences between the groups in terms of gravidity, mode of delivery, timing of delivery, proportion of stillbirths and blood transfusion. A significantly higher proportion of multiparous women presented with abruption when compared to healthy controls (P=0.039). There was a significantly higher proportion of women who delivered preterm (P=0.026) and through caesarean section (P=0.001). The incidence of stillbirth (P=0.000) and blood transfusion (P=0.000) was significantly higher among cases when compared to controls.

**Table 1:** Demographic and Obstetric characteristics of the study population. * Chisquare Test.

Characteristics	Cases, N (%)	Controls, N(%)	P Value
Age (Mean ± SD) (years)	26.82 ± 5.50	28.82 ± 4.88	0.057
Religion			
Hindu	46 (92)	41 (82)	0.137
Muslim	04 (08)	9 (18)	
Residence			
Urban	38 (76)	43 (86)	0.202
Rural	12 (24)	7 (14)	
Occupation			
Housewife	43 (86)	41 (82)	0.585
Working	07 (14)	9 (18)	
Socio-economic Status			
Lower Class	47 (94)	41 (82)	0.065
Lower Middle class	03 (06)	9 (18)	
Diet			
Vegetarian	41 (82)	41 (82)	1.00
Nonvegetarian	9 (18)	09 (18)	
Gravidity			
Primigravida	14 (28)	24 (48)	0.039
Multigravida	36 (72)	26 (52)	
Mode of delivery			
Caesarean	22 (44)	7 (14)	0.001
Vaginal	28 (56)	43 (86)	
Timing of delivery			
Preterm	19	9	0.026
Term	31	41	
Stillbirth			
Yes	19 (38)	2	0.000
No	31 (62)	48	
Blood transfusion			
Yes	48 (96)	4	0.000
No	02 (04)	52	

**Figure 1:**
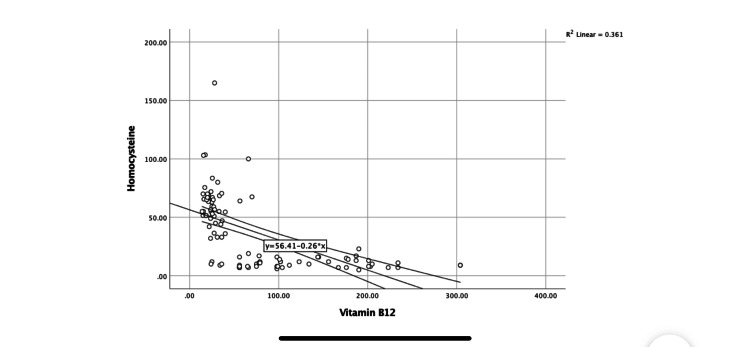
Correlation of serum homocysteine level with serum vitamin B12 level (Pearson correlation=-0.601, P=0.000).

Mean serum micronutrient levels among the cases and controls are presented in Table 2. The mean concentration of homocysteine and vitamin B12 have significant difference between the groups. The serum level of homocysteine is significantly correlated with serum vitamin B12 level (Pearson correlation=-0.601, P=0.000) (Figure 1). However, folic acid concentration between the groups remains comparable.

**Table 2:** Serum micronutrient levels among the study population. * Independent T test

Serum Micronutrient level	Cases (Mean ± SD)	Controls (Mean ± SD)	P value
Serum vitamin B12 (picogram/ml)	27.15 ± 11.63	125.84 ± 70.48	0.000
Serum homocysteine (micromole/L)	62.57 ± 21.79	10.78 ± 3.82	0.000
Serum folic acid (nanogram/ml)	47.98± 13.15	43.79 ± 13.72	0.123

Mean plasma level of vitamin B12 (pg/ml) varied significantly among women delivering by caesarean and vaginal delivery while other values remain insignificant (Table 3).

**Table 3:** Association of serum homocysteine (µmol/L), folic acid (ng/ml) and vitamin B12 (pg/ml) with obstetric outcome. a: Mann Whitney test b: T test

Parameters	Mean ± SD Homocysteine (µmol/L)	P value	Mean ± SD Folic acid (ng/ml)	P value	Mean ± SD Vitamin B12 (pg/ml)	P value
Time of birth						
Preterm (n=32)	61.02 ± 18.12	0.839^a^	46.94 ± 13.85	0.46^b^	28.73 ± 13.44	0.485^a^
Term (n=18)	65.34 ± 27.51		49.83 ± 11.95		24.33 ± 6.87	
Mode of Delivery						
Caesarean (n=22)	65.1 ± 28.08	0.845^a^	48.7 ± 15.4	0.734^b^	23.34 ± 6.74	0.006^a^
Vaginal (n=28)	60.59 ± 15.46		47.41 ± 11.34		30.14 ± 13.75	
Status of birth						
Live birth (n=31)	62.84 ± 23.89	0.92^a^	49.18 ± 15.45	0.351^b^	26.44 ± 11.69	0.406^a^
Stillbirth (n=19)	62.13 ± 18.46		46.03 ± 8.13		28.32 ± 11.75	

## Discussion

Homocysteine is found in low concentration in all tissues under normal conditions [1]. However, its raised level is cytotoxic that adversely affects the endothelial system including the placenta. The hypothesis that hyper-homocysteinemia is a risk factor for vascular disease was proposed approximately 50 years ago by McCully [8] and is considered to be associated with abruption.

Our study found a high level of serum homocysteine and low level of vitamin B12 in all cases of abruptio placentae when compared to controls with comparable folic acid levels between the groups.

Choudhary et al. in their cohort analysis of 7587 participants found no significant association of maternal plasma homocysteine concentration with placental abruption [9]. However, Goddijn-Wessel way back in 1996 reported association of hyperhomocysteinemia with the obstetric complication [10]. Both these studies did not determine vitamin B12 levels. We determined levels of both of them and found low level of vitamin B12 in women with abruption. Hence, vitamin B12 as significant determinant of the occurrence of abruption cannot be ruled out and the predictability of serum homocysteine alone for the obstetric catastrophic needs to be evaluated further.

Homocysteine level is elevated in vitamin B12 deficiency, and the homocysteine levels can predict vitamin B12 deficiency with a sensitivity of greater than 95% [11-13]. This is in tune with our finding of significant negative correlation between the serum levels of homocysteine and vitamin B12. The pathophysiology of raised homocysteine consequent to low vitamin B12 initiating the occurrence of placenta mediated pregnancy complications has a number of theories. Nelen et al. suggested that homocysteine inhibits a number of mechanisms which promote anticoagulation by endothelium of blood vessels. It also prevents the binding of tissue plasminogen activator to endothelium by inhibiting the fibrinolytic properties of the endothelial surface [14]. Placental abruption occurring probably from vascular catastrophe found synchronously with infarction of placenta substantially affects the prognosis of maternal and foetal wellbeing. The infarction being essentially due to occlusion of spiral artery in the myometrium or decidua [14]. Hyper-homocysteinemia presumably considered a weak prothrombotic factor exerts its effects through a number of hypothesised mechanisms like exaggerated expression of tissue factor, accelerated platelet activity and generation of thrombin, elevated activity of factor V, diminished fibrinolytic processes, and injury to vascular endothelium [15]. Beyond these assumptions of thrombotic mechanisms, imprecise molecular actions like oxidative stress, hypomethylation of DNA, and proinflammatory effects may have a role in the pathogenesis. This prothrombotic property of homocysteine is implicated in the occurrence of strokes and myocardial infarction too [15].

In a systematic review analysing the role of folic acid and homocysteine in the occurrence of placental abruption, folate deficiency was implicated as a risk factor with pooled odds ratio of 25.9 ( 95 CI 0.9-736.3) [16]. However we failed to demonstrate low levels of folic acid in our cases of abruption probably because in Indian population, folic acid intake is good as Indian diet is rich in fruits and green leafy vegetables. Besides, folic acid is supplemented along with iron preparation in all antenatal women in accordance with national protocols to prevent anaemia in them [17].

However, deficiency of vitamin B12 is rather common in developing countries like India with a prevalence of 43-47% in young adults [18]. Since vitamin B12 is derived from animal sources, and Indian population being mostly vegetarian such finding of high prevalence needs attention. The custom of boiling milk further decreases the dietary intake of vitamin B12 even in vegetarian Indians. Hence, Indians may be born with low vitamin B12 status due to low maternal levels and then continue to have low status due to vegetarian diet and malabsorption due to tropical sprue. This has led to intergenerational inheritance of low vitamin B12 stores. Hemodilution in pregnancy further declines its levels with prevalence reported being as high as 40-60% in various studies [19,20].

When considering the age of individuals with abruption, there is discordance in findings of our study with respect to others. Zu et al. found homocysteine levels first decrease and then increase in both gender remaining lowest between 30-50 years of age and then increasing significantly after 50 years of age [21]. When considering the relation of abruption with age, advanced maternal age (>35 years) is found to be a risk factor [22]. In a meta-analysis by Martinelli et al., women > 40 years of age were found to have 1.44 times odds of developing abruptio compared to women in the 35-40 years group [2]. However, we found most of the women were between 20-30 years (68%) of age and the overall distribution of elderly pregnant women in our study was less (10%). The proportion (62%) of multiparous women among cases was high. Our findings are similar to Ananth et al. [23] who observed that the incidence of placenta previa and abruptio placentae abruption was high in young multiparous women. They hypothesised that the higher risk of uteroplacental bleeding disorders in younger women with high order parity could be due to shortened interpregnancy intervals affecting the uteroplacental bleeding disorders apart from many unmeasured confounders like income, education, nutrition etc.

The obstetric outcome, substantially affected by the levels of homocysteine, folic acid and vitamin B12 level is proven by studies. In a retrospective cohort study of 11,549 pregnant women by Yuan et al., it was found that an imbalance in the levels of serum folate and vitamin B12, depicted by deranged serum folate to vitamin B12 ratio before delivery can predict adverse pregnancy events posing higher risks for intrahepatic cholestasis of pregnancy, pregnancy-induced hypertension and large-for-gestational-age [24]. A significant difference in the serum levels of the vitamins among the cases and controls reinforces this finding in our study too. A higher proportion of caesarean delivery among cases was found which is often necessary in the setting of placental abruption to limit further oxygen deprivation to the foetus, to reduce blood loss for the mother and, in extreme cases, to prevent the death of the foetus, the mother, or both. The incidence of preterm delivery (64%) was too significantly high among cases. Spontaneous preterm birth in abruption occurs due to bleeding and separation of the placenta, which irritates the uterine lining and stimulates contractions. Thirty-eight percent of women delivered stillborn babies in our study attributable to asphyxia in the setting of abruption.

Out of the many causes of abruptio placentae, the micronutrient association to its occurrence and severity has not been researched extensively till now. The index study sheds light on this aspect and highlights the relationship and outcome. This indeed forms the premise for a future prospective robust observation incorporating all potential factors presumed to affect this obstetric complication. The genetic polymorphism in enzymes of homocysteine, folic acid and vitamin B12 was not determined.

## Conclusions

Hence we conclude Vitamin B12 and homocysteine are significant determinants of abruptio placentae in pregnant women. Animals being the source of Vitamin B12, the vegetarian Indian pregnant population remains at high risk of developing complications consequent to its deficiency. Recommendations for supplementation with the vitamin in high risk Indian population shall go a long way to provide considerable protection against the hazards of abruption, preterm delivery, foetal growth restriction and foetal death occurring due to raised homocysteine.
